# Urgent call to action: Supporting Morocco in the aftermath of the recent earthquake

**DOI:** 10.7189/jogh.13.03065

**Published:** 2023-12-20

**Authors:** Malik Olatunde Oduoye, Latif Ur Rehman, Samuel Chinonso Ubechu, Lawal Abdulkareem, Marina Ramzy Mourid, Hamza Irfan

**Affiliations:** 1 Department of Medical Research Circle, Bukavu, Democratic Republic of Congo; 2 Department of Allied Health Sciences, Iqra National University, Peshawar, Pakistan; 3 School of Public Health, Yale University, New Haven, Connecticut, USA; 4 College of Health Sciences, Usman Danfodiyo University, Sokoto State, Kaduna State, Nigeria; 5 Faculty of Medicine, Alexandria University, Alexandria, Egypt; 6 Department of Public Health and Community Medicine, Shaikh Khalifa Bin Zayed Al Nahyan Medical and Dental College, Lahore, Pakistan

The 2023 earthquake in Morocco caused significant damage to the country’s infrastructure. The Global Disaster Alert and Coordination System (GDACS) reported that most of the damage had been sustained by key infrastructure [1]. However, according to Stratfor, the Moroccan earthquake crisis can expectedly severely harm the economy by 2024 [2]. Meanwhile, Capital Economics (CE) suggests that earthquake crises are unlikely to impact the Moroccan economy majorly [3]. Local authorities and humanitarian organisations are grappling with a formidable challenge due to the profound impact of earthquakes on infrastructure. The main objective of this article is to underscore the pressing requirement for immediate action in dealing with the aftermath of the earthquake crisis, outline the hurdles encountered, and emphasize the significance of interventions related to mental health and psychosocial support (MHPSS). Furthermore, this article pleads for assistance from the international community and MHPSS organisations and accentuates the critical role of knowledge dissemination for future disaster preparedness and recovery endeavors.

## OVERVIEW OF THE MOROCCAN EARTHQUAKE OF 2023

The devastating Moroccan earthquakes of 8 September 2023 profoundly impacted the country. The first earthquake struck at 11 pm Moroccan local time, with a magnitude of 6.8, followed by 4.9 aftershocks 19 minutes later [1]. These earthquakes caused significant structural damage to buildings and the country’s infrastructure, resulting in over 2600 injuries and 2500 fatalities [4]. The epicenters of the earthquakes were located in the High Atlas Mountains of Morocco [5,6]. According to the US Geological Survey (USGS), the earthquake was an unusual occurrence in western Morocco, and the affected regions comprised the Middle East and North Africa (MENA) regions of Morocco [7]. However, the impact was severe, and the Moroccan nation required immediate interventions to address the aftermath crises. These urgent interventions included food, clean water, shelter, and medical care facilities with the support of local authorities and various international humanitarian organisations to restore the normalcy required in the affected areas.

## URGENT INTERVENTIONS NEEDED

An earthquake marked by sudden and extensive disruption to the human ecosystem requires immediate external assistance and support. Natural disasters, such as earthquakes, landslides, floods, and typhoons, exert substantial pressure on hospital systems because of their capacity to generate a high volume of injuries within a condensed time frame [8,9]. Simultaneously, these natural disasters inflict significant disturbances upon hospital infrastructures, impeding their operational capabilities. For instance, a seismic event of magnitude 7.6 in Turkey in 1999 resulted in approximately 50 000 injuries in Izmir and wrought extensive damage to ten major hospitals, necessitating the relocation of most patients from these health care facilities [10].

Following an earthquake, addressing the physical needs of affected individuals is paramount. One of the primary physical needs that emerges prominently following an earthquake is the provision of shelter. This is due to the often-extensive damage or outright destruction of residences resulting from seismic events [11]. Immediate efforts must be directed toward offering secure and stable housing solutions to mitigate exposure to the elements and potential harm. Another critical physical need is access to nutrition. This is particularly important when an earthquake disrupts the regular food and clean water supply. Preventing malnutrition and dehydration through the prompt distribution of essential provisions is vital for the affected population [12].

Timely and appropriate medical care is also essential to address the injuries incurred during the earthquake. This encompasses not only the treatment of physical trauma but also the management of medical conditions that the disaster may exacerbate. Identifying and mitigating hazards within the post-earthquake environment is important to ensure safety [13]. Immediate assessment and correction of safety concerns are crucial for preventing further physical harm. In addition, trauma associated with earthquakes often leads to crushing injuries and injuries affecting both bony and soft tissues. Consequently, there is a demand for the expertise and services of physical therapists [14].

The present earthquake in Morocco could have many public health implications, ranging from psychological problems such as depression and posttraumatic stress disorder (PTSD), etc. to aid the victims, as well as predisposing children to become orphans, children on the street, or children on the streets. In addition, a high vulnerability to child abuse, gender-based violence (GBV), sexual exploitation, and child trafficking could occur due to the earthquake in Morocco. Pregnant women in Morocco could be at risk of miscarriages, stillbirths, and other obstetric problems. The economy of Morocco could also drastically decline, leading to an increased poverty rate in the country. Furthermore, more social vices and crimes could occur, especially among youths. Therefore, all these aforementioned health implications call for urgent interventions.

## CHALLENGES FACED AND ITS IMPORTANCE

Responding to the recent earthquake in Morocco has presented numerous challenges for local authorities and humanitarian organisations. With a staggering death toll exceeding 2800 and thousands injured, the scale of the disaster has stretched the resources and capabilities of responders [15]. In addition to these difficulties, the region has a history of colonialism, authoritarianism, economic disparities, corruption, and human rights concerns that have contributed to the region's vulnerability [16]. Additionally, the predominant use of traditional construction materials such as mud bricks and rough wood has made it extremely difficult to locate survivors, as these structures tend to collapse entirely, leaving minimal air pockets. Moreover, the rugged terrain of the Atlas Mountains, where the earthquake occurred, poses logistical challenges, with blocked roads obstructing aid delivery. According to Al Jazeera’s reporter Hashem Ahelbarra, the teams trying to assist after the earthquake in Morocco are facing very tough challenges. Ahelbarra talked about how difficult these problems are. He said that the rough land and big rocks on the roads make it hard to bring aid to the people who need it [17].

## CALL FOR ACTION FOR SUPPORT

Morocco faced immense challenges in the aftermath of the earthquake, with infrastructure damage, injuries, and trauma prevalent. In the aftermath of the recent earthquake in Morocco, there is a critical need for the provision of mental health and psychosocial support (MHPSS). The catastrophic event, characterized by a substantial loss of life, widespread injuries, and extensive property damage, has left a significant portion of the affected population dealing with acute psychological distress. The psychological trauma of such a disaster can manifest as anxiety disorders, depression, and posttraumatic stress disorder (PTSD). Considering the challenges posed by the earthquake’s impact on access to affected regions, it is imperative to establish timely and accessible mental health interventions. To address these pressing issues, the international community must extend its support. Doing so can foster goodwill, strengthen international relations, and demonstrate solidarity despite adversity. Collaboration between local authorities and humanitarian organisations is essential to develop a comprehensive framework that includes psychological counselling, support group initiatives, and community-based psychosocial programs. These efforts are essential for reducing the long-term psychological impact and enhancing the overall resilience and recovery prospects of earthquake survivors in Morocco.

Over the years, losses caused by natural disasters such as earthquakes and flooding have continuously increased despite the growing research on natural risks [18]. Experts in hazard control explicitly mention that improved knowledge is insufficient to reverse the upward trend in disaster statistics. These experts ponder how knowledge is used in hazard management [18]. It has been found that a better appraisal of the actual results of applying the best available knowledge in the best possible way at the community and other levels is needed. Additionally, there is a need to build upon past achievements to create a better understanding of natural hazards [18].

The occurrence of natural hazards, such as the recent earthquake in Morocco, cannot be avoided, at least in the present state of our knowledge and ability. However, Oduoye et al. revealed that the impact of such events on humans and their properties can be reduced to reasonable levels [19]. In the case of Morocco, it is imperative that earthquake risk or damage reduction concerns all the measures needed before the next earthquake, which could reduce the consequences of such an event [19]. We commend the humanitarian assistance provided by the international community, philanthropists, and celebrities worldwide, especially football star Cristiano Ronaldo, who offered his 174-room hotel as a shelter for Morocco’s earthquake victims [20]. This demonstrates positive awareness of the earthquake in Morocco. On this note, emergency response teams from all the neighboring countries of Morocco, Algeria, Spain, and Western Sahara [21], including the world’s powerful countries like the United States of America, the United Kingdom, China, Germany, Russia, Japan, and Korea, should be dispatched as soon as possible to the incident scene. We urge these countries to provide medical aid, supplies, food, and clothing for earthquake victims in Morocco [19]. These countries must consider the affected Moroccans as refugees in their respective countries by providing them with adequate food, clothing, and shelter during this difficult time.

We recommend that the government of Morocco collaborate with external organisations such as the World Medical Association (WMA), the Food and Agriculture Organization (FAO), the United Nations Children’s Fund (UNICEF), the United Nations Educational, Scientific and Cultural Organization (UNESCO), and the United Nations Population Fund (UNFPA) on disaster preparedness and recovery in Morocco. A qualitative distinction between different levels of understanding provides a sound basis for researchers to better relate to policymakers and practitioners in Morocco’s disaster risk reduction (DRR) domain [18]. The government of Morocco should also institute adequate protection by the military to safeguard the most vulnerable people, including children, pregnant women, and the elderly in Morocco, against other violence such as rape, trafficking, abuse, and exploitation, as it has been proven that a time of natural hazard is an opportunity for social vices and exploitation [18,19].

## CONCLUSIONS

Despite the recent earthquake in Morocco, the international community has an opportunity to demonstrate its compassion and solidarity. By providing financial aid, medical assistance, humanitarian support, and mental health services, we can help Morocco recover and rebuild. Let us stand together, extend a helping hand, and make a meaningful difference in the lives of those affected by this tragedy. We believe all global health efforts should be adequately harnessed during this difficult time for Morocco.
